# Notes on Three Years’ Ovariotomy Work in the Samaritan Free Hospital: Eighty-two Cases without a Death

**Published:** 1888-09

**Authors:** George Granville Bantock

**Affiliations:** Surgeon to the Samaritan Free Hospital


### Selections.

### NOTES ON THREE-YEARS' OVARIOTOMY WORK IN THE SAMARITAN FREE HOSPITAL: EIGHTY-TWO CASES WITHOUT A DEATH.

By GEORGE GRANVILLE BANTOCK, M. D., F. R. C. S. Ed.,
Surgeon to the Samaritan Free Hospital.

[From the *British Medical Journal,* June 30, 1888. Extract from the Author's Reprint.]

From the middle of April, 1885, to the corresponding date this year, I have performed eighty-two ovariotomies in the Samaritan Free Hospital, and all the patients have recovered.

On the whole, the cases have been of a rather severe character, as the following details will show. Thus, in more than one-half—forty-seven—there were adhesions properly so-called; in small proportion the adhesions involved the parietes only, or omentum only; in large proportion both these structures were involved at the same time; in considerable number the tumor was adherent to the pelvic organs to a varying extent, and in a few instances to the intestines also. In one of parietal adhesions only, the patient had just completed seven months of her first pregnancy, in the course of which she had been twice tapped. (This case was published in the *Journal,* February 11, 1888, p. 296.)

Excluding all these cases of adhesions proper, there were six cases in which there was no pedicle, and the tumor had to be enucleated. In one of these the cyst was in a state of suppuration; in another, nine stout ligatures were required to arrest the bleeding from the torn structures left after removal of a tumor involving the left broad ligament and left side of the uterus, and in the last case of this kind operated upon, the last but one of this series, the operation was so severe that when the patient was put into bed, the pulse could scarcely be counted at the wrist. In yet another the tumor apparently involved both ovaries, and when the enucleation was completed, the uterus was so bereft of support, in addition to being extensively injured, that I was compelled to remove it also at the level of the internal os in my usual way.

Thus, of the eighty-two cases, there were adhesions or their equivalent in about two-thirds, or fifty-four, leaving twenty-eight in which there were no adhesions, and there was a pedicle capable of being treated by ligatures. In one of these twenty-eight cases there was a serious complication in the form of a large umbilical hernia, measuring over four inches across. After removing an ovarian tumor of ten pounds (right side), I slit open the hernial sac by continuing the abdominal incision, applied several ligatures to, divided the adherant omentum, and then dissected off the peritoneum from the various pouches, cutting away the redundant and thinned skin and peritoneum. In closing the wound I first brought the peritoneal edges together in the region of the hernia by closely applied silk-worm gut sutures, of which the ends were cut off short, and then the skin and intervening tissues by deep sutures running along under the raw surface, and not going through the peritoneum. The remainder of the wound, in which the fatty layer measured nearly two inches in depth, midway between the umbilicus and pubes, was closed in the usual way. Convalescence was uninterrupted, and the hernial sac was completely obliterated.

As further evidence of the severity of the cases, I may point out that the drainage-tube was used in more than one-half, or forty-five, and in these, with few exceptions, the peritoneum was washed out with warm water.

In thirty-four cases the second ovary was more or less diseased, and was accordingly removed. It is sometimes very difficult to decide whether the ovary is sufficiently diseased to justify its removal. In a large majority of the cases there was no difficulty whatever, and a very recent case induces me to believe that I shall find less difficulty in the future by requiring less distinct evidence than hitherto. I removed an ovarian tumor of nine and one-half pounds, eleven years ago. The other (left) ovary appeared to be quite healthy, and this view may be said to have been confirmed by the fact that within two years the patient gave birth to twins (one of each sex). But there is pretty conclusive evidence that it did not long remain healthy, for there is a history of gradual increase in the size of the abdomen for several years, and I have recently removed this second ovary, forming a tumor of eighteen pounds. In this instance I was asked why I had not removed the second ovary at the first operation.

In all there were nine cases of dermoid tumors, and in one ot these both ovaries were diseased, the one containing skin and hair, and the other containing teeth. This is the second instance of double dermoid disease I have seen. The other was a private case. In only four of these cases were there adhesions; the remaining five were free, though in one the pedicle was twisted. The absence of adhesions in this instance was to be explained by the fact that the circulation through the pedicle had not been sufficiently interfered with to set up degenerative changes in the tumors.

In eight cases the pedicle was twisted, and three of these belonged to the dermoid variety. In all but one there were more or less extensive adhesions, and in these seven the peritoneum was washed out and drained.

There were five cases of ruptured cyst, in all of which the whole peritoneal cavity was thoroughly washed out and a drainage-tube was used.

In three cases the disease was malignant, and in two of these the malignancy was recognized at the time of the operation. In the first case the patient died in about fourteen months with extensively disseminated cancer. In the second the patient made a rapid and uninterrupted recovery, leaving the hospital on the twentieth day. In a few weeks more a small tumor formed in the region of the pedicle, painful and tender, without any rise of temperature or increase of pulse, and accompanied with rapid emaciation, and she died on July 6th. In the third case there was great œdema of the lower extremities, gastric irritation with frequent vomiting, and considerable distension of the abdomen by free fluid. At the operation twenty-five pints of free fluid were removed together with a sarcoma of the left ovary weighing six pounds, and another of the right ovary weighing about three-fourths of a pound, and the pelvic and lumbar glands were already infected. The peritoneum was well washed out and drained, and she recovered without a bad symptom. The disease, however, made rapid progress, though there was no more ascites; the gastric irritation, relieved by the operation, returned in a few weeks along with the œdema of the lower extremities, and she died in seven weeks.

All these eighty-two operations were done without the use of any so-called antiseptic substance. Warm water—not specially prepared—was freely employed in washing out the peritoneal cavity whenever the contents of the cyst had escaped into it, either by spontaneous rupture before operation, or when, through the breaking down of extensive adhesions, there was any effusion of blood and serum amongst the intestines, rendering the "toilet of the peritoneum" very difficult by any other method. In all these cases the drainage-tube was also employed. In not one single instance was any opium or alcohol administered.

In the year 1882 a comparison was instituted between the results obtained in the Samaritan Free Hospital under the Listerian and non-

Listerian methods, the latter being wholly confined to my practice. That comparison was very much to my disadvantage, and deductions were drawn which even the results of the following year tended to upset, and which those of succeeding years have actually tended to reverse. Again, that comparison was made on the results of only one year. A comparison of results extending over several years will be a more reliable guide, and I now present those for the three years 1885, 1886 and 1887, in the following tables:

| Dr. Bantock's Cases. | | | Listerian Cases (Three Operators). | | |
|---|---|---|---|---|---|
| Total. | Recovered. | Died. | Total. | Recovered. | Died. |
| 79 | 78 | 1 | 121 | 110 | 11 |

But in September, 1887, Mr. Doran abandoned the Listerian and adopted my method of practice, and if his three cases be added in the one table, and subtracted in the other, the following result is obtained:

| Dr. Bantock's Cases. | | | Listerian Cases (Three Operators). | | |
|---|---|---|---|---|---|
| Total. | Recovered. | Died. | Total. | Recovered. | Died. |
| 82 | 81 | 1 | 118 | 107 | 11 |

If, again, the results for the three years over which the preceding eighty-two cases extend, namely, from April, 1885, to April, 1888, be taken, the following is the result:

| Dr. Bantock's Cases. | | | Listerian Cases. | | |
|---|---|---|---|---|---|
| Total. | Recovered. | Died. | Total. | Recovered. | Died. |
| 82 | 82 | 0 | 116 | 104 | 12 |
| Plus Mr. Doran's Cases. | | | Minus Mr. Doran's Cases (Three in 1887 and one in 1888). | | |
| Total. | Recovered. | Died. | | | |
| 4 | 4 | 0 | | | |

Giving eighty-six cases without a death, as against 116 cases with twelve deaths, or a mortality of 10.34 per cent.

